# Review of the possible association between thyroid and breast carcinoma

**DOI:** 10.1186/s12957-018-1436-0

**Published:** 2018-07-05

**Authors:** Liangbo Dong, Jun Lu, Bangbo Zhao, Weibin Wang, Yupei Zhao

**Affiliations:** Department of General Surgery, Peking Union Medical College Hospital, Chinese Academy of Medical Science and Peking Union Medical College, Beijing, 100730 People’s Republic of China

**Keywords:** Thyroid cancer, Breast cancer, Iodine, Sodium iodide symporter, Thyroid hormone, Thyroid hormone receptor, Gonadal hormone, Obesity, Radioactive iodide therapy

## Abstract

**Background:**

Thyroid and breast cancer are two of the malignant diseases with highest incidence in females. Based on clinical experience, breast and thyroid cancer often occur metachronously or synchronously. Therefore, thyroid and breast cancer might share some common etiological factors. The relationship between these diseases has attracted substantial attention, and because these two glands are both regulated by the hypothalamic-pituitary axis, such a relationship is not surprising. A study of this relationship will be useful for obtaining a better understanding of the mechanism by which these two malignancies co-occur.

**Main body:**

This study reviewed the progress in research on the roles of iodine intake, folate metabolism, obesity, gonadal hormones, and thyroid hormone in thyroid and breast cancer. These studies evaluating the etiological roles of these factors in linking breast and thyroid cancer might also improve our understanding and identify new therapeutic approaches, such as sodium/iodide symporter-mediated radioiodine therapy and thyroid-stimulating hormone receptor antagonists, for breast cancer. In addition, some specific treatments for each cancer, such as radiotherapy for breast cancer or radioactive iodine therapy for thyroid cancer, might be risk factors for secondary malignances, including breast and thyroid cancer.

**Conclusions:**

Studies of the precise relationship between the co-occurrence of breast and thyroid cancer will certainly improve our understanding of the biological behaviors of these two malignancies and direct evidence-based clinical practice.

## Background

The thyroid and mammary glands are both regulated by the hypothalamic-pituitary axis. Based on clinical experience, patients were diagnosed with breast and thyroid cancer metachronously or synchronously more frequently than expected by accident. However, their mechanisms of action remain unknown. Are these two common malignancies in women related? Several studies have detected a possible association between these malignancies. Jee Hyun An, Yul Hwangbo, et al. carried out a retrospective case-control study that suggested that the overall risk of second primary thyroid cancer (TC) or breast cancer (BC) is significantly increased in patients who previously had BC or TC, respectively [1]. Previous studies have also confirmed this finding [2–5]. As the genesis and development of TC and BC are associated, researchers must investigate the causes of these types of multiple primary tumors (MPTs). This study reviewed the recent progress in research on the roles of iodine intake, folate metabolism, obesity, gonadal hormones, thyroid hormone, and signaling pathways in thyroid and breast cancer.

## Iodide

### Iodide, iodine transport, and breast cancer

**Fig. 1:**
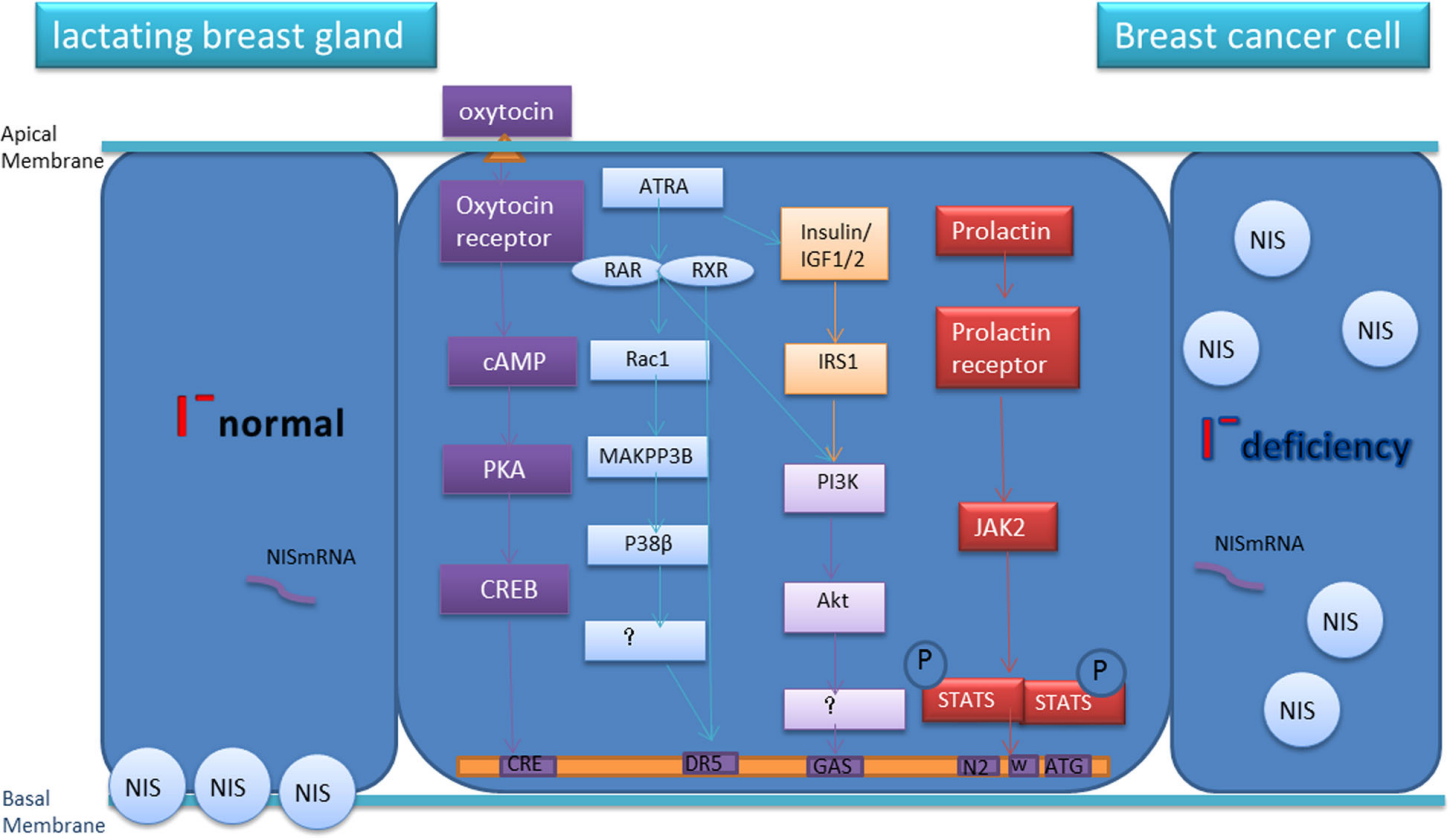
Regulation and expression of the NIS gene in breast cancer. In breast cancer cells, the NIS protein is predominantly expressed in the intracellular space, whereas the protein is located on the basolateral membrane in lactating mammary glands. Mislocalization of the NIS protein may lead to a disparity between the NIS expression level and observed radioiodide uptake

There is overlap between TC and BC regarding the uptake and utilization of dietary iodine. Several studies have focused on the role of iodine in BC. Hypothyroidism and low iodine intake may be important preventable etiological factors in estrogen-dependent tumors of the breast, uterus, and ovary. Iodine supplementation may lead to a decreased incidence of these cancers in future generations. The sodium/iodine symporter (NIS), a large integral plasma membrane glycoprotein that mediates iodide uptake, is expressed at its highest levels in the thyroid and lactating breast [6–8]. Over 40 years ago, BC tissues were shown to take up radioactive iodine; in contrast, uptake does not occur in normal, non-lactating breast tissues [9]. 25 years later, NIS mRNA was first detected in breast cancer specimens [10]**.** In 2001, Moon DH, Lee SH, et al. studied the correlation between the expression of the human NIS mRNA and the uptake of 99mTc-pertechnetate in 25 breast tumors. However, compared with NIS mRNA expression, the level of iodide uptake was relatively low [11]. Later, in 2003, the NIS protein was shown to be predominantly expressed in the intracellular space, whereas NIS in lactating mammary glands is located on the basolateral membrane [6]. Therefore, researchers have hypothesized that the mislocalization of NIS protein may lead to the disparity between the NIS expression level and observed radioiodide uptake. Based on endocrinological studies, iodine deficiency may stimulate the gonadotrophin secretion and then result in a hyperestrogenic state, which possesses characteristics of relatively high production of estrone and estradiol and a relatively low estriol to estrone and estradiol ratio. This alteration in endocrine state may increase the risk of BC [12]. In addition, strategies that increase dietary iodine intake may reduce the risk of BC [13] **(**Fig. 1). Conversely, excess iodide intake also plays an unfavorable role in BC by stimulating ER-α transcriptional activity [14]. Malya FU et al. reported a high urine-iodine concentration (UIC) in a significantly larger portion of BC patients than controls [15]. Others have also indicated that NIS can be used as an objective criterion for predicting the sensitivity of luminal B and basal BC subtypes to neoadjuvant chemotherapy, which will improve treatment outcomes in this group of patients [16].

### Iodine, iodide transport, and thyroid cancer

Iodine deficiency is a well-established risk factor for the development of TC, [17] and iodine supplementation has been carried out in most areas with endemic goiter. Chronic iodine deficiency may have some protective effects on females, but no equivalent studies have detected its effect on males [18]. As shown in the study by Anne-Catherine Gerard et al., iodine deficiency induces the expression of the vascular endothelial growth factor (VEGF) mRNA in both normal thyroid cells and TC cells. This effect lasts longer in thyroid carcinoma cell lines, suggesting impairment of downregulation mechanism. Moreover, the iodine deficiency-induced VEGF expression partially depends on hypoxia-inducible factor-1 (HIF-1) instead of on reactive oxygen species. Thus, iodine deficiency may provide an angiogenic environment for abnormal proliferation of TC cells [19]. By contrast, excess iodine has also been connected with an increased incidence of papillary thyroid cancer (PTC). After the implementation of universal salt iodization in China in 1996, the incidence of goiter was reduced by almost 50%; however, the incidence of TC is steadily increasing [20]. Haixia Guan from Johns Hopkins Hospital and others have focused on this issue and initiated a study measuring and comparing the mutation of the T1799A BRAF in 1032 patients from five regions in China with different levels of iodine intake. As high iodine intake contributes to the occurrence of the T1799A BRAF gene mutation, it may be a risk factor for the development of PTC [21]. According to a cross-sectional study from South China, the mean UIC, an index for evaluating the nutritional status of a population, is significantly higher in patients with thyroid nodules than in healthy individuals; of these thyroid nodules, approximately 5–15% were malignant, i.e., TC [22]. However, the conclusive link between excess iodide and TC remains unclear. Others also believe that this increase in the incidence is mainly due to advances in diagnostic technology.

In conclusion, dietary iodide deficiency and intracellular iodide deficiency caused by mislocalization of NIS may play a role in the carcinogenesis of TC and BC. Iodine deficiency may cause DNA damage in the thyroid gland and promote cancer [23]. Moreover, studies on the role of iodide in BC provide promising therapeutic approaches. For instance, NIS-mediated radioiodine therapy for estrogen receptor-negative BC has been studied in more detail [24], and Kelkar MG recently reported that histone deacetylase inhibitors (HDACis) as modulators of NIS expression can significantly increase NIS expression but do not alter its intracellular localization. Thus, there is a promising future for NIS-mediated radioiodine therapy [25]. Kelkar MG, first highlighted the role of p53 as a negative transcriptional regulator of human functional NIS gene expression in BC, providing important perspectives into the promising clinical use of NIS-mediated radioiodine therapy, which may significantly impact a patient with a mutant versus wild-type p53 profile [26].

## Sex hormones, reproductive factors, and thyroid cancer

Hormones induce oncogenesis by promoting cell proliferation, an essential component of carcinogenesis. In addition, their important roles have been well studied in BC and prostate cancers [27]. TC exhibits a great gender disparity; it is 2.9-fold more common in women than in men. A significant difference between men and women is their sex hormones and the influences of these hormones on multiple organs and systems. The fluctuating levels of sex hormones during a woman’s menstrual cycle and pregnancy have been hypothesized to be the root cause of the gender disparity in PTC [28]. Because TC is highly prevalent in fertile women, hormonal and reproductive factors may also be involved in its incidence [29]. As shown in the 1993 study by Inoue et al., estradiol increases proliferation of estrogen receptor (ER)-positive PTC, supporting the hypothesis that estrogen promotes the proliferation of this type of disease [30]. Estradiol also alters the expression of estrogen receptor subtypes in TC cell lines [31–33]. Guia et al. investigated the expression of estrogen receptor α (ERα) and progesterone receptor (PR) in female and male patients with PTC and connected their levels with the clinical manifestation and molecular features. ERα and PR were detected in 66.5 and 75.8% of cases, respectively. Their expression significantly relates to a larger tumor size and higher prevalence of local metastases. Furthermore, the “receptor conversion” (variation in receptor status in primary and metastatic BC) phenomenon was first observed in thyroid cancer and has subsequently been reported in BC [29]. Estrogen promotes growth through classical genomic and non-genomic pathways, which are mediated via the membrane-bound ER. This receptor is correlated to the Aurora-like serine/threonine kinase (APK) and phosphoinositol 3-kinase (PI3K) tyrosine kinase signaling pathways. However, in contrast to other carcinomas, detailed information about this regulatory mechanism has not been reported for TC [34]. As shown in the study by Rajoria et al., estrogen is associated with increased adherence, invasion, and migration of TC cell lines. Thus, the higher occurrence rate of TC in women could possibly due to the expression of a functional ER, which participates in cellular processes that contribute to the enhanced mitogenic, migratory, and invasive potential of thyroid cells. These findings will promote the future development of anti-estrogenic therapies targeting neoplasm invasion and migration, thus reducing the tendency to metastasize [35]. Membrane-bound ER is linked to the mitogen-activated protein kinase (MAPK) and PI3K tyrosine kinase signaling pathways, and in PTC, these pathways may be activated by either chromosomal rearrangement of the tyrosine receptor kinase (TRKA), RET/PTC genes, or a BRAF mutation. Additionally, these pathways may be stimulated by high estrogen levels in females. Furthermore, estrogen regulates angiogenesis and metastasis, which are crucial to the outcomes of TC [34].

## Thyroid hormones (THs), TH receptor β1 (TRβ1), antibodies, and breast cancer

### THs

Thyroid hormones exert diverse critical biological effects on the growth, differentiation, metabolism, and physiological function of almost all human tissues, including the mammary gland [36, 37]. However, the correlation between thyroid function and BC is uncertain. Søgaard M, Farkas DK, et al. conducted a nationwide cohort study, which included 61,873 women with hypothyroidism and 80,343 women with hyperthyroidism, in which the women with hyperthyroidism had greater risk of developing BC, and the women with hypothyroidism had a slightly decreased incidence of BC [38]. By evaluating a large cohort of women, Journy NMY et al. also reported that the risk of BC mortality elevated in women with hyperthyroidism with hyperthyroidism after 60 years of age [39]. Others have already focused on the mechanism underlying this phenomenon. As reported in the study by Moretto FC, De Sibio MT, et al., triiodothyronine (T3) may induce the expression of HIF-1 and transform growth factor alpha (TGF) in the MCF7 breast cancer cell line. These factors are related to the genesis and development of BC. Moreover, thiiodothyronine (T3) exerts this effect by activating PI3K [40]. As shown in the 2002 study by Sumi Dinda et al., T3 may regulate the cell cycle progression and proliferation of T47D cells (an estrogen-responsive human ductal carcinoma cell line that expresses detectable levels of ER) by increasing the p53 levels and inducing pRb hyperphosphorylation via a common mechanism involving the ER and T3 receptor-mediated pathways [41]. Additionally, Hall LC et al. supported this hypothesis and reported that T3 induced activation of ER-mediated gene expression and promoted the proliferation of MCF7 cells. Although the effects were weaker than those induced by E2, T3 may play roles in BC development and progression [42]. In the 2010 study by Tosovic A et al., the T3 levels were shown to be positively and dose-dependently related to the BC risk in postmenopausal women [43]. The 2005 study by P.P. Saraiva also supported the above conclusion that high T3 levels in postmenopausal women are positively and dose-dependently correlated with the risk of BC. However, this situation was observed in postmenopausal women who had a significant increase in their thyroid/estradiol ratio. These phenomena may suggest that the imbalance between E2 and T3 promotes the genesis and development of BC [44]. In contrast, hypothyroidism has been associated with a decreased risk of BC [45]. However, the association between TH and BC is currently controversial, and conclusive evidence is lacking. In a study by Johannes LP et al., hypothyroidism and low-normal free T4 (FT4) levels were reported to be correlated to an increased risk of BC in postmenopausal women [46]. Overall, further studies are needed to investigate the precise association between thyroid function and BC.

### TR

TR belongs to the nuclear hormone receptor superfamily, similar to classical biomarkers of BC, such as ER and PR. Its exact role in the genesis and progression of BC has been known for years. Several scientists investigated this question in 2014. As demonstrated in the study by Sobine Heublein, Doris Mayr, et al., TRs may be an interesting biomarker and prognostic factor for patients with BRCA1-associated BC. TRβ positivity may be positively related to the five-year or overall survival of BC patients, whereas TRα has opposing actions [47]. Jeon won Park et al. studied the mutation of TRβ, which is considered to have oncogenic activity. According to previous studies, TRβ1 may function as a tumor suppressor. However, TRβ1 expression is silenced by several mechanisms, such as hypermethylation of the promoter region and a microRNA-mediated regulatory mechanism. Additionally, TRβ1 mutations also cause it to lose its tumor suppressor function. In addition to the C-terminal frameshift mutation PV, some additional sequences in the C-terminal regions of TRβ1, such as Mkar, Mdbs, and AM, also exhibit oncogenic activity, promoting cell proliferation and suppressing differentiation and apoptosis [48]. Some researchers in China have reported that aberrant TRβ1 expression and mutations are associated with the genesis and development of BC in the Chinese population [49]. Nonetheless, the exact role of TR in the genesis of BC remains unclear. Further studies are needed to identify a new biomarker of BC and new strategies for developing targeted therapy.

As discussed above, altered TH function and dysfunction of TR contribute to the increased incidence of BC. Other factors, such as autoimmune antibodies, may also play a role in its genesis [50]. Autoimmune thyroid diseases, such as Graves’ disease, are characterized by increased levels of thyroid peroxidase antibodies (TPOAbs) and thyroglobulin antibodies (TgAbs). These two autoimmune antibodies have a well-established association with BC. In addition, Pawel S. et al. in 2013 showed the TSH receptor (TSHR) antibody to be a positive determinant of BC and the only positive determinant in the analysis of age-matched patients. Thus, TSHR antagonists may potentially play a prophylactic role in BC, and additional clinical research is advisable [51].

## Others

The genesis and development of both TC and BC are quite complicated. Some other factors may play a role in the co-existence of TC and BC. Radioactive iodine therapy is a routine therapy for differentiated TC in Western countries, and it is increasingly being used in China. As mentioned above, mammary gland cells also express NIS, thus, the breast tissue may also absorb radioactive iodine, and the absorption of a high dose of radioactive substances may induce carcinogenesis [52]. The results of a retrospective single-center study in Portugal suggest that the risk of developing second primary cancer is increased after radioactive iodine therapy, particularly for activities > 200 mCi [53]. However, because of the lack of statistics, this hypothesis requires further support. Additionally, in a 2015 long-term follow-up study, radioactive iodine (RAI) therapy did not significantly increase the occurrence and recurrence of subsequent BC [54]. In 2015, Zhang YJ et al. from Peking Union Medical College conducted a meta-analysis that included 6 cohort studies, involving 17,914 patients. The results suggested that the risk of secondary primary BC in TC survivors treated with RAI did not increase compared with TC survivors not treated with RAI [55]. Folate metabolism, which plays an essential role in DNA synthesis, is another important aspect of carcinogenesis, and it was recently shown to be involved in the increased incidence of TC and BC. As shown in a study by Zara-Lopes T, an alteration in the methylenetetrahydrofolate reductase (MTHFR) gene that participates in folate metabolism, C677T, is significantly associated with the increased incidence of thyroid and breast cancer. These factors may be used as potential predictive and prognostic markers for both types of cancer [56]. Obesity and a higher cancer risk have a well-established, strong association; weight, weight gain, and obesity are responsible for approximately 20% of all malignant neoplasms. TC and BC are no exception [57]. A 2016 study in Korea reported a positive association between a high body mass index (BMI) and TC incidence, and prevention efforts, such as weight gain control, may reduce the burden of TC [58]. Yunji Hwang et al. conducted a large-scale case-control study and suggested that middle-aged adults who gain weight have a higher risk of developing PTC. Although this study has some limitations, such as recall and detection bias, the results still suggest that weight gain control can decrease the incidence of TC [59]. A study in France also supported this hypothesis. Clavel-Chapelon F et al. identified a significant dose-dependent association between the risk of developing TC and BMI, particularly in women who gained weight from menarche to adulthood [60]. Meanwhile, the role of obesity or a high BMI in the development of breast cancer has been well-known for years [61, 62]. Moreover, weight loss interventions are recommended for patients with BC [63].

## Conclusions

In summary, the etiologies of thyroid and mammary gland cancers share common features, such as iodine intake and transport and the levels of thyroid function, TH receptors, obesity, and sex hormones. Factors that contribute to the initiation of TC, such as low dietary iodine, hypothyroidism, and other thyroid disorders, may also contribute to the increased risk of BC. Some factors, such as estrogen and reproductive factors that play well-established roles in BC initiation, may be associated with TC. These studies may help to explain why these two cancers occur metachronously or synchronously more frequently than would be expected by chance. Moreover, studies on the commonalities between these two cancers may provide new prophylactic therapeutic strategies and early diagnostic methods, such as anti-estrogen therapy for thyroid carcinoma and anti-TSHR therapy for breast carcinoma. Although further theoretical and clinical studies are still needed before these treatments are applied in the clinic, future clinical applications are promising. The precise relationship between the co-occurrence of breast and thyroid cancer remains controversial and inconclusive, yet studies of their co-occurrence will certainly improve our understanding of the biological behaviors of these two malignancies and direct evidence-based clinical practice.

## Abbreviations

BC: Breast cancer
MPTs: Multiple primary tumors
NIS: Sodium/iodide symporter
PTC: Papillary thyroid cancer
TC: Thyroid cancer
TH: Thyroid hormone
TRβ1: TH receptor β1
TSH: Thyroid stimulating hormone
TSHR: Thyroid-stimulating hormone receptor
